# Study protocol: Return to Country, an Australia-wide prospective observational study about returning First Nations renal patients home

**DOI:** 10.1136/bmjopen-2024-095727

**Published:** 2024-11-24

**Authors:** Yomei Jones, Mandy Truong, Cecilia Preece, Alan Cass, Fiona Heerink, Stephen McDonald, Scott Jones, Andrew John Mallett, Sandawana William Majoni, Cherian Sajiv, Khalil Patankar, Eddie Mulholland, Solomon Woldeyohannes, Paul Lawton

**Affiliations:** 1 Wellbeing and Preventable Chronic Diseases, Menzies School of Health Research, Casuarina, Northern Territory, Australia; 2 School of Nursing and Midwifery, Monash University, Clayton, Victoria, Australia; 3 Australian Institute of Family Studies, Southbank, Victoria, Australia; 4 Menzies School of Health Research, Charles Darwin University, Darwin, Northern Territory, Australia; 5 ANZDATA Registry, South Australia Health and Medical Research Institute, Adelaide, South Australia, Australia; 6 Faculty of Medicine, University of Adelaide, Adelaide, South Australia, Australia; 7 Department of Nephrology, John Hunter Hospital, New Lambton Heights, New South Wales, Australia; 8 Department of Renal Medicine, Townsville Hospital and Health Service, Townsville, Queensland, Australia; 9 James Cook University Faculty of Medicine Health and Molecular Sciences, Townsville, Queensland, Australia; 10 Department of Renal Medicine, Royal Darwin Hospital, Casuarina, Northern Territory, Australia; 11 Flinders University Northern Territory Medical Program, Darwin, Northern Territory, Australia; 12 Department of Renal Medicine, Alice Springs Hospital, Alice Springs, Northern Territory, Australia; 13 Department of Renal Medicine, Royal Perth Hospital, Perth, Western Australia, Australia; 14 Sacred Business Services, Darwin, Northern Territory, Australia; 15 School of Translational Medicine, Monash University, Melbourne, Victoria, Australia; 16 Menzies School of Health Research, Casuarina, Northern Territory, Australia

**Keywords:** Quality Improvement, Dialysis, Health policy, Health Equity, Health Services Accessibility, End stage renal failure

## Abstract

**Introduction:**

In Australia, kidney failure treatment disparities exist between Aboriginal and/or Torres Strait Islander (First Nations) and non-First Nations people. Despite persistent calls from First Nations patients with kidney failure, they are less likely to have treatment that allows them to live at home.

**Methods and analysis:**

This is a prospective, multicentre study based in Australia. The aim of the study is to characterise the socioeconomic, environmental, health service and biomedical factors driving the health outcomes and patterns of health service utilisation experienced by First Nations patients and investigate whether health service changes to address these identified barriers can achieve higher rates of renal replacement therapy at home on country. This will be achieved by mixed-methods data collection at health service (audit and process data), staff (surveys and qualitative interviews) and patient (survey testing, feedback sessions, health outcomes) levels. A process evaluation will identify barriers and enablers to health services changes in relation to cultural safety. Baseline and follow-up data will be compared to assess the extent to which health services change their service delivery and the impact on health outcomes for First Nations patients with kidney failure. Qualitative and quantitative data will be integrated to provide an in-depth understanding of project outcomes and impacts.

**Ethics and dissemination:**

This study is funded by the National Health and Medical Research Council of Australia (GNT1158075). Ethics approval has been obtained so far from the Human Research Ethics Committee (HREC) of the Northern Territory Department of Health and Menzies School of Health Research (2019-3530), Far North Queensland HREC (2023/QCH/99606 (Nov ver 4)-1732), the Central Adelaide Local Health Network HREC (2023/HRE00209), the Aboriginal Health Council of South Australia (AHREC Protocol #: 04-23-1078), the Aboriginal Health and Medical Research Council of New South Wales (AH&MRC HREC reference: 2230/24) and the Far North Queensland Human Research Ethics Committee (FNQ HREC reference: HREC/2023/QCH/99606 (Nov ver 4)-1732). Study participants, policy makers and community organisations will be provided with updates of study findings. Dissemination of study findings will be through peer-reviewed publications and conference presentations.

**Trial registration number:**

ACTRN12623001241628.

**STRENGTHS AND LIMITATIONS OF THIS STUDY**

- This multicentre prospective study is led by a team of First Nations and non-First Nations researchers, with extensive First Nations community consultation.
- A focus on service-level factors (ie, policies, procedures and practices, staff beliefs and behaviours) is a particular strength of the study and will provide understanding of barriers and facilitators related to organisational change.
- This study is grounded in First Nations patient experiences and outcomes: culturally safe practices are defined by the patients and their communities.
- This study includes a participatory action component with lead clinician investigators as co-champions driving change together with patients and local communities.
- Evidence from this study will inform both national guidelines for the care of First Nations patients in tertiary care settings, and local improvements to care at involved sites.

## Introduction

 Kidney failure, when dialysis or a kidney transplant is required to maintain life, is a rapidly increasing global health and healthcare burden.^1^ Worldwide, access to care for patients at risk for kidney failure or in need of treatment is most challenging for those in low- and middle-income countries.^1^ While kidney failure incidence is comparatively low in high-income countries such as Australia, disparities exist between Aboriginal and/or Torres Strait Islander (First Nations) and non-First Nations people.^2^

First Nations Australians have a higher incidence of kidney failure compared with non-First Nations Australians. They have at least six times the age-standardised incidence of renal replacement therapy (RRT) of non-First Nations Australians.^2 3^ Although only 3.2% of the Australian population,^4^ almost 1 in 10 patients commencing RRT each year in Australia identify as First Nations.^2^ Among adults aged 25–64 and people from rural and remote areas, rates are up to 15 times higher.^5 6^ More than half of First Nations Australian kidney failure patients come from remote or very remote areas. (In comparison, only 1.1% of non-First Nations kidney failure patients come from remote or very remote areas of Australia.)^7^

Additionally, First Nations patients experience very different patterns of RRT. Where most non-First Nations patients with kidney failure receive treatment that allows them to live at home, the majority of First Nations patients still receive urban or regional facility-based treatment.^2 8^ Community-based dialysis or a kidney transplant allows a patient to return to live in their community.^2^ However, uptake of these modalities (community nurse-facilitated haemodialysis, self-care haemodialysis, peritoneal dialysis and kidney transplantation) among First Nations Australians continues to be low.

For over 25 years, First Nations health organisations and patients in rural and remote Australia have persistently called for more responsive treatment, closer to home, for First Nations peoples with end-stage kidney disease.^9 10^ Community-led advocacy groups have continued this call in more recent years. A national meeting of First Nations kidney failure patients in September 2017 renewed this message.^11^ Over the last 15 years, substantial progress has been made in expanding and decentralising haemodialysis care across remote Australia.^12^ Nevertheless, most treatment is still provided as haemodialysis in nurse-facilitated centres in major or regional towns, rather than at home in remote communities.^6^

Various barriers to treatment at home on country (Being at home on ‘country’ refers to the lands, waterways and seas to which First Nations peoples are connected. The term contains complex ideas about law, place, custom, language, spiritual belief, cultural practice, material sustenance, family and identity (AIATSIS 2021 https://aiatsis.gov.au/explore/welcome-country).) have been reported: remoteness, cost, the presence and severity of comorbid medical conditions, housing and environmental issues, low levels of health literacy, poor practitioner–patient communication, lack of engagement, cultural factors, practitioner-, service- and system-level biases.^13^,^15^ The physical and psychosocial demands of dialysis treatment for First Nations patients are accentuated by family separation and ineffective communication between patients and their healthcare providers.^10^ First Nations patients are often young and socially isolated when relocated from rural and remote communities to urban settings for regular dialysis treatment. Both kidney transplantation and self-care dialysis provide a superior quality of life at costs cheaper than facility-based dialysis for non-First Nations Australians. However, self-care treatment options are underused for First Nations people with kidney failure.^2 16^

International literature has demonstrated that both educational attainment and levels of health literacy correlate with access to RRT modalities such as kidney transplantation or self-care dialysis.^17 18^ First Nations RRT patients often feel that they have been excluded from information and feel poorly equipped to seek it.^19^ Qualitative research has documented concerns about poor communication between First Nations dialysis patients and non-First Nations health professionals.^1119^,^22^ Ineffective communication with healthcare practitioners is associated with poorer outcomes for First Nations patients, including confusion and frustration^19 21^; discharge against medical advice^23^ and distrust of healthcare providers.^24 25^ Interpreters for First Nations patients remain grossly underused.^26^ Furthermore, experiences of racism in the healthcare sector have been reported by First Nations Australians^27^ and have been explored qualitatively as a key reason for avoiding or refusing healthcare.^28^

Culturally safe healthcare provision has been promoted to improve healthcare outcomes for First Nations people globally.^29 30^ Cultural safety refers to: ‘the ongoing critical reflection of health practitioner knowledge, skills, attitudes, practising behaviours and power differentials in delivering safe, accessible and responsive healthcare free of racism’.^31^ Ultimately, First Nations individuals, families and communities are the ones who decide what is culturally safe.^32 33^ Moreover, organisational- and structural-level policies, practices and procedures need to promote culturally safe care to address institutional and systemic racism.^34^ The assessment of systemic and/or institutional levels of cultural safety affecting First Nations Australians is currently more challenging than the measurement of practitioner-level cultural safety. Systematic reviews of cultural safety training for health practitioners have found some improvements in practitioners’ awareness, knowledge and behaviours; however, the majority of these studies use self-reported surveys.^35 36^ While frameworks^37 38^ exist that seek to explain the role of service-, system- and societal-level biases in poorer outcomes,^39^ their measurement is still largely conceptual.^33^ Few studies have examined the effect of service- and system-level interventions,^40^ thus more high-quality research is needed and in different health settings and locations to examine the effect of system-level interventions on the provision of culturally safe healthcare.

### Study aim and objectives

**Figure 1:**
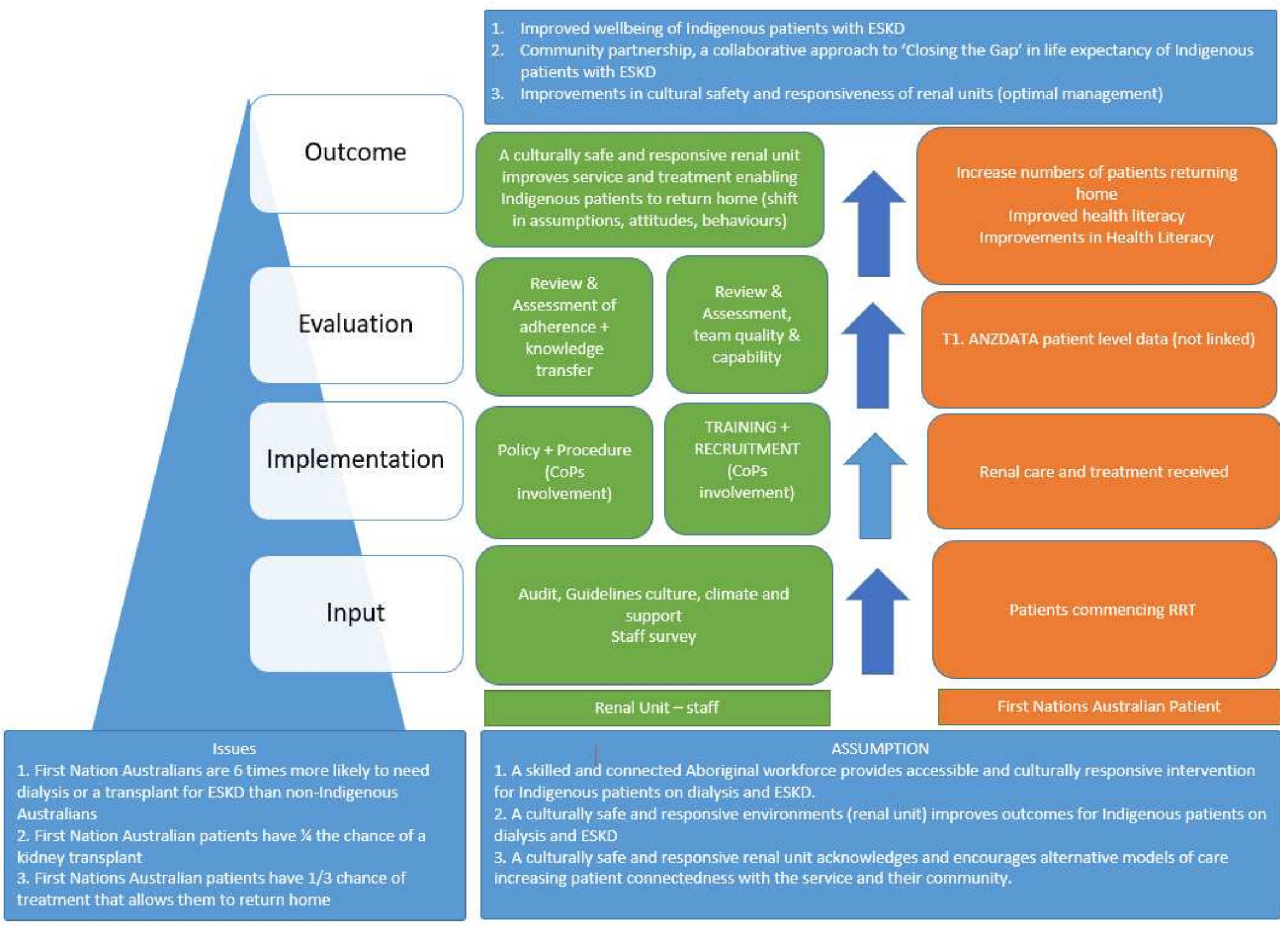
Return to Country—study design and programme logic.

The aim of the study is to characterise the socioeconomic, environmental, health service and biomedical factors driving the health outcomes and patterns of health service utilisation experienced by First Nations Australians and investigate whether health service changes to address these identified barriers can achieve higher rates of RRT closer to home. The study’s hypothesis is that service-level attributes are more important than patient factors in the association with rates of ‘returning home’ models of care, and that some service-level attributes are able to be modified to increase rates of ‘returning home’ care. An overview of Return to Country (RTC) programme logic, including hypotheses and aims, can be found in figure 1.

Objectives are:

1. To test the feasibility, acceptability and appropriateness of a patient survey adapted for First Nations peoples (both in English and several First Nations languages).
2. To understand the factors associated with treatment disparities (ie, in relation to transplantation and dialysis treatment) experienced by First Nations patients compared with non-First Nations patients.
3. To assess the level of renal health services’ cultural safety from the patient perspective.
4. To identify the barriers and enablers to organisational change in relation to the provision of culturally safe care to First Nations patients with kidney failure.
5. To assess and evaluate the extent to which renal health services change their delivery of care to First Nations patients with kidney failure following an assessment of their level of cultural safety.
6. To assess the impact of the renal health service-based changes on health outcomes for First Nations patients with kidney failure.

## Methods and analysis

The RTC study is a prospective, multicentre study based in Australia.

### Study setting

14 tertiary renal health services (of approximately 90) throughout Australia have been invited to take part in the RTC study. These services coordinate care for at least five new First Nations patients starting RRT each year on average, and together they care for up to 90% of First Nations patients starting RRT nationally each year. Of those, four services care for approximately 60% of all incident First Nations RRT patients, and a further four care for an additional 15–20% of incident First Nations RRT patients.^2^

### Study design

**Figure 2:**
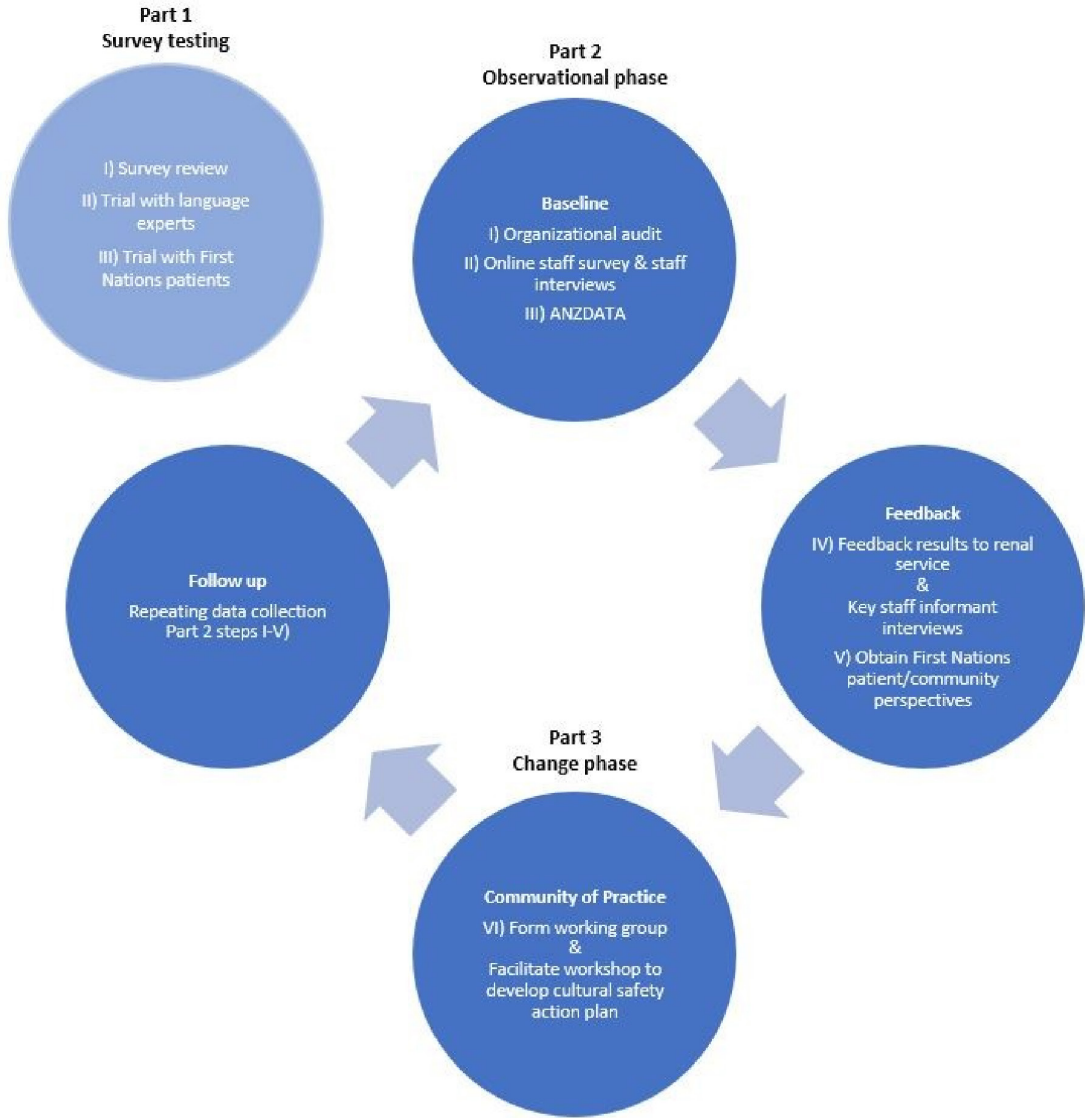
Return to Country—study components.

RTC is a mixed-methods project consisting of three parts as shown in figure 2.

### Part 1: survey testing

The objective of the first part of RTC is a test of the feasibility and appropriateness of a patient survey process to understand First Nations patient reported experiences and outcomes, including the acceptability, translatability and content validity of survey items. The RTC patient survey has five domains: ‘Demographic and Housing’; ‘Wellness’; ‘Experiences of Racism’; ‘Health Literacy’ and ‘Care and Treatment’. Survey testing will be conducted in several services located in the Northern Territory, Queensland and New South Wales to assess where differences exist for First Nations patients that predominate speaking First Nations language versus those that predominately speak English.

#### Data collection

**Figure 3:**
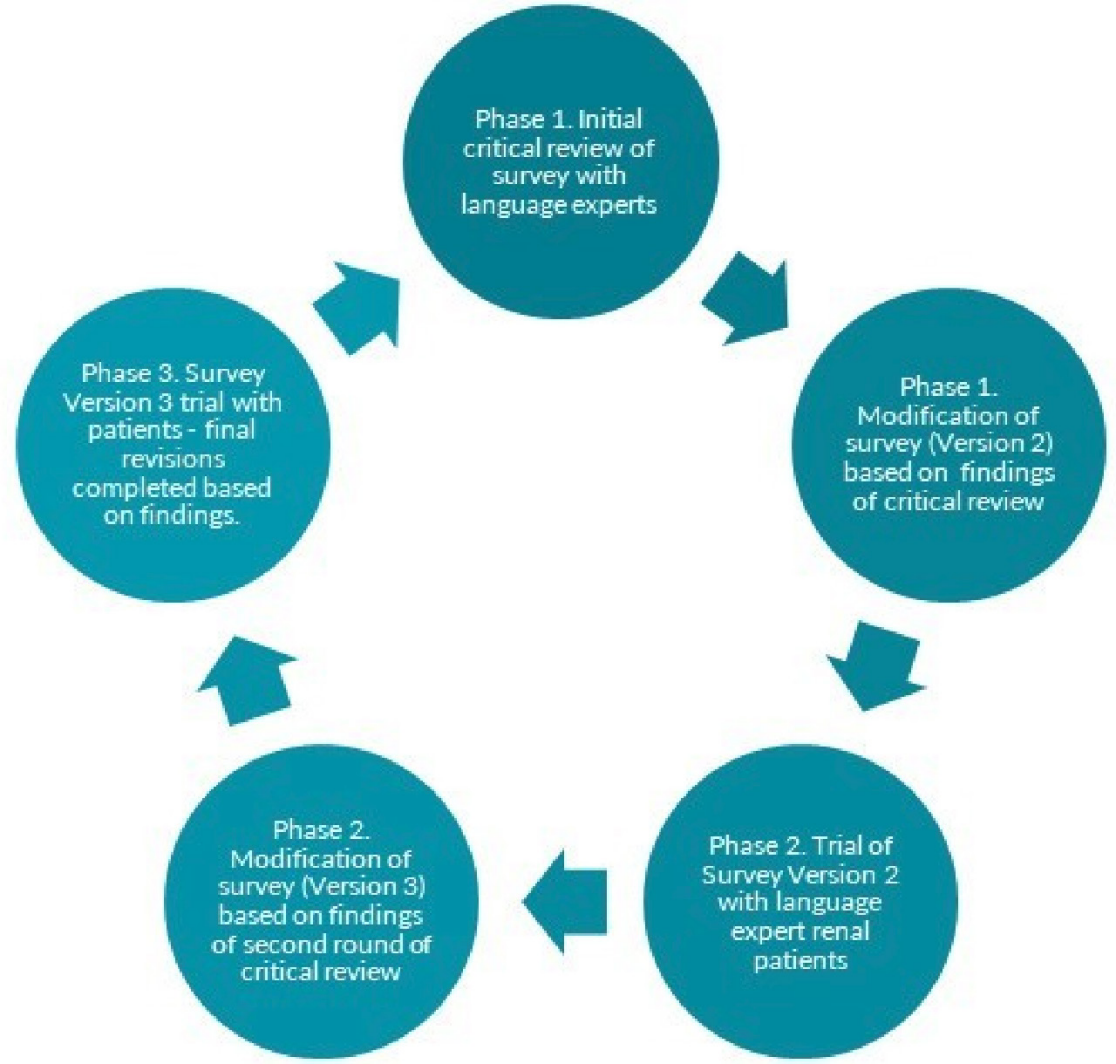
Phases of survey development.

Patient participants will be identified by a First Nations community representative and approached by the local research team. Part 1 consists of three steps, which can be found in figure 3. First, plain English survey items will be critically reviewed and modified with a sample (n=15) of language experts (including some patients receiving dialysis). Second, this version will be trialled with participants using adapted video reflexive ethnography and/or an integrated process of reflection and critical analysis as the survey is administered. The review process in the first and second step involves extensive discussion with participants and does not reflect the time and attention demands that will be involved in the typical administration of the survey. Therefore, tolerability will be explored in step III when the survey is administered as intended with recently commenced patients, within 12 months after commencing RRT. This process will be conducted in several services located in the Northern Territory, Queensland and New South Wales to assess whether differences exist between different First Nations language groups. All activities will be audio- or video-recorded.

#### Data analysis

Data from the first two steps will be translated, transcribed and thematically analysed using an iterative and inductive process while exploring factors that influence acceptability, translatability and feasibility. This process will inform alterations to survey administration procedures and revisions of survey items. Content validity of survey items will be explored through comparison with participant responses during survey administration and comparison with peer-reviewed qualitative research of renal patient experiences. Step III will analyse tolerability by comparing patient-identified priority topics from in-depth interviews after survey administration with their survey responses. Findings of Part 1 will inform the development and use of surveys in a culturally safe way with First Nations peoples and determine whether the patient survey is used in other renal health services.

### Part 2: observation

The second part of RTC is an investigation of the level of cultural safety of renal health services. A mixed methods approach will be used to identify and examine patient-, provider-, service- and system-level factors that impact First Nations renal patients’ ability to obtain treatment closer to home.

#### Data collection

**Figure 4:**
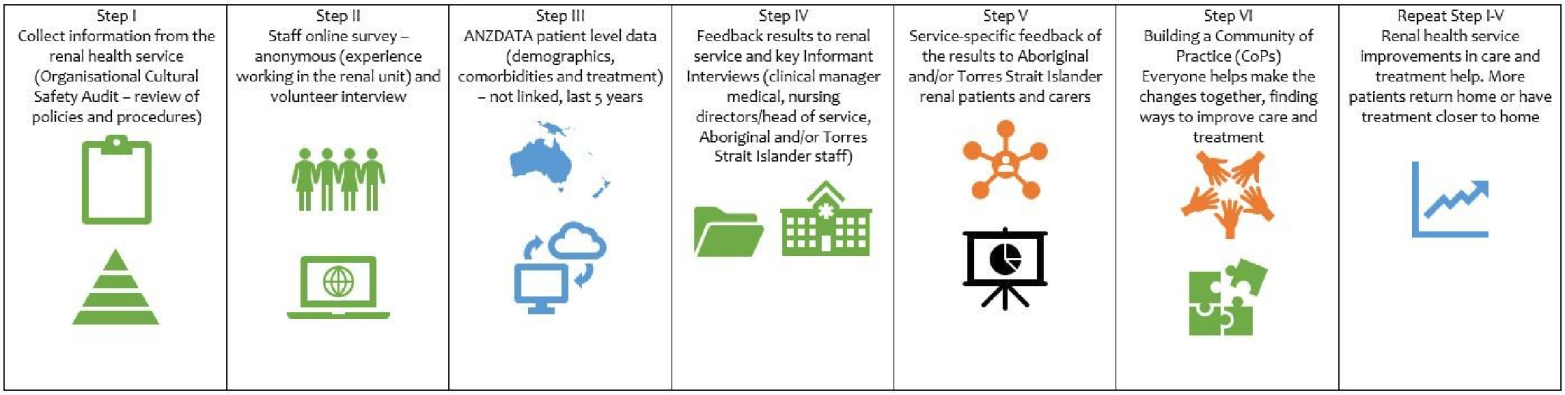
Return to Country—data collection steps.

Part 2 consists of five steps, which are shown in figure 4.

##### (I) Organisational Cultural Safety Audit

Renal health services will take part in an Organisational Cultural Safety Audit of policies, practices and procedures. The audit tool will highlight areas performing well and areas for improvement. It can be used to monitor change over time. The audit tool is based on the National Safety and Quality Health Service Standards (NSQHSS) User Guide for First Nations Health^38^ and organisational health literacy-related indicators and a section from the Congress of Aboriginal and Torres Strait Islander Nurses and Midwives (CATSINAM) framework.^41^ Senior clinic management staff of each renal service will be invited to complete the audit for their service and study personnel will be available to assist on request.

##### (II) Staff survey and interviews

All staff at each site will be invited to complete an anonymous online staff survey, including medical doctors, registered nurses, allied health staff and any other non-clinical staff employed in the renal health service. The survey asks about their opinions and experiences working in the renal service and looks at patient-, staff-, service- and system-level factors that may impact cultural safety in renal services. Informed by the work of Skinner *et al*,^42^ the survey includes elements from the Modern Racism Scale^43^ and the Attitude Towards Indigenous Australians Scale,^44^ as well as questions about intercultural communication.^45^ Each service will have its own unique link to enable individual participants’ responses to be grouped by service level. If requested, paper copies of the staff survey can be provided. Staff may also elect to participate in a one-to-one interview with study personnel to explore staff survey domains in more detail. The staff survey will be administered by the Social Research Centre, based in Melbourne, Victoria, Australia.

##### (III) ANZDATA registry data

Data will be requested from ANZDATA to provide a 5-year summary of the incidence and prevalence of each dialysis and transplant modality for First Nations and non-First Nations patients at each service. ANZDATA data include centre-specific reports with deidentified patient data related to postcode at entry and at census dates, comorbidities, treatment modalities and outcomes (at entry and throughout the treatment journey) and treatment centre. All renal units in Australia and New Zealand contribute data to the national renal (ANZDATA) registry.

##### (IV) Feedback to staff and key informant interviews

The results from the audit, staff surveys and interviews and ANZDATA registry data will be reported back to senior medical and nursing clinicians. This provides a foundation for renal health service critical reflection and considers areas for improvement. Open group feedback sessions for renal service staff will provide an opportunity for further discussion of results and to identify areas that could be strengthened to improve organisational cultural safety.

Semistructured qualitative key informant interviews will be conducted with key staff at the renal health service to understand the capacity to improve the provision of culturally safe care to First Nations patients. An estimated five key staff can include the Head of Unit, Nursing Director, Aboriginal Health Practitioner/Worker, Social Worker, Aboriginal Liaison Officer, transplant coordinator or other allied health professionals. The interview guide will be informed by findings from the organisational audit and staff survey and include topics related to organisation readiness and implementation barriers and facilitators from the Consolidated Framework For Implementation Research.^46^

##### (V) Talking circles with patients

Feedback of findings will also be provided to First Nations patients to obtain perspectives. First Nations patients with kidney failure and their carers will be invited to attend the talking circles, where results of their service’s organisational audit and staff survey will be presented. Patient sample selection being voluntary and therefore a ‘convenience’ sample of those interested; care will be taken to ensure that transport challenges, disability and/or language needs are met through study funding. With individual patient consent, two talking circles will be conducted over two consecutive days (to accommodate differing prescribed dialysis schedules). The discussions will be recorded and analysed to develop an understanding of patient and carer perspectives about the cultural safety of their renal service.

### Data analysis

#### (I) Organisational Cultural Safety Audit

The organisational policies and procedures audit tool will be completed by renal unit management staff, assisted by RTC study personnel if requested. The completed audit domains will be reviewed by RTC study personnel to determine the extent to which current unit/service documents (including policies, procedures and plans) meet the RTC cultural safety guidelines across the seven domains. For each renal health service, study personnel will provide an audit score over seven domains based on the completed and supporting information provided by senior service staff. The seven domains of assessment are ‘Working in partnership’; ‘Addressing health needs of First Nations people’; ‘Cultural awareness and cultural safety’; ‘Implementation and monitoring’; ‘Governance and identifying priorities’; ‘Welcoming environment’ and ‘Organisational Health Literacy’. A level of cultural safety will be assigned by RTC project personnel to the service for each domain using the categories: ‘action strongly recommended’, ‘should be strengthened’ and ‘performing well’.

#### (II) Staff survey

Concluding each survey period (typically 2 months), deidentified staff survey data will be collated by the Social Research Centre and sent to study personnel using secure methods. A descriptive analysis of cross-sectional staff survey data will first be conducted to describe participant characteristics and provide frequency distributions to each question. For each service, a score across all participants and responses will be derived in total for each domain. Multi-level statistical models incorporating aggregate service-level data (aggregate staff survey tool scores, for example) and individual patient-level clinical and sociodemographic data (clustered at the service level) will be developed. Differences in service-level scores will be compared between baseline and follow-up, and across the different sites.

#### (III) ANZDATA registry

Data fields will be requested that enable the development of a summary of the incidence and prevalence of each dialysis and transplant modality at each service, an analysis of the probability of incident patients to receive an RRT modality that could enable patients to return home, and an analysis of the probability of incident patients to be recorded as receiving care at the same residential postcode as identified at the start of their treatment (particularly those from remote or very remote areas).

Standardised Incidence Ratios for each centre will be determined as follows. Separately, logistic regression (to estimate the outcome of community-based treatment at 1 year after starting treatment) and Poisson regression (to estimate the outcome of the number of days recorded for each patient receiving community-based treatment within the first year) will be used. Standard bivariate and stepwise techniques will be used initially for each fixed effects model to determine explanatory patient-level variables of greatest utility; subsequently, a two-level random-effects model that includes useful fixed-effects patient-level variables and random effects for each treatment centre and a random slope for First Nations status will be used to estimate the expected result for each centre for each outcome.^47 48^

During each bootstrap replicates (500 boot samples of size N where N is the size of the original data): (i) we’ll fit a random effects regression model with patient-level fixed effects, and centres as random effects and First Nations status as a random slope, (ii) the weighted predicted probability of the ‘RTC’ outcomes of interest will be computed using size of centres to total size of the bootstrap data as weights, (iii) the logarithm of the observed to the weighted predicted ratio will be computed for each of the K centres, resulting in k centre-specific risk adjusted standardised incidence ratios (log-SIR); (iv) we’ll construct 95% and 99% FDR for each centre’s log-SIR using normal-theory bootstrap methods. That is, for each centre, we’ll compute the SD of the estimated log-SIR 500 bootstrap replicates. This quantity will enable a calculation of the estimate of the SE of the estimated log-SIR. A 95% FDR for each centre’s log-SIR will then be computed as the estimated log-SIR from the original data. Therefore, the (1−α)% FDR will be computed for each centre as follows:

FDR=Log SIR±Z(α/2)×the bootstrap estimate of the SE of log-SIR.^49^

Ultimately, as noted above, it may be possible to include additional centre-specific data as noted above (including summary staff survey scores and organisational cultural safety audit scores) as explanatory random effects in the statistical models that predict the ‘RTC’ quantitative outcomes of interest.

The Log-SIR along with 95% and 99% FDRs will be presented using funnel plots. The funnel plot shows the log-SIR for each centre against their effective sample size which is a measure of the variability of the SIR estimate. The effective sample size is defined as a measure of the variability of the log-SMRs (logarithm of the standardised mortality ratios) for each centre relative to the total variability of all log-SMRs.^47 48^ It is used as the x-axis for the funnel plots to give smooth curves for the FDR lines.

Both STATA V.18 and R software will be used for quantitative data analysis. It is expected that open source R software code will be published alongside the results of analysis, with each centre publicly deidentified (but privately identified for reports to each centre’s executive, staff and patients about their centre’s involvement in the project). Data shared by ANZDATA for this project will not be able to be published, in line with the standard ANZDATA data release agreement, but could be made available by ANZDATA on request.

#### (II), (IV) and (V) Qualitative data analysis

Because text and image data will be dense and rich, the qualitative data analysis will require sequential steps to be followed, involving multiple levels of analysis. Qualitative data from staff interviews, key informant interviews and feedback circles will be translated (as needed) and transcribed verbatim—with or without annotations for behaviour (eg, pausing, laughing, crying); field notes will be typed up, visual and audio material will be catalogued and sorted to read or look at. This will be a collaborative process involving First Nation researchers and will yield a general sense of the information, providing an opportunity for reflection before a process of coding using both inductive and deductive coding approaches. NVivo software will be used to manage and analyse the qualitative data where appropriate. The representing of the description and themes will be represented by using narrative passages to convey the findings for discussion, as in grounded theory. There will be separate analysis for Study Parts 1–3.

For example, to understand the factors associated with renal health disparities that is, in relation to transplantation and dialysis treatment experienced by First Nations patients compared with non-First Nations patients, a range of methods will be used to enable triangulation of data across multiple sources and perspectives. Thematic and content analysis of service staff perspectives about the organisational audit data, staff survey data and ANZDATA outcomes data will potentially highlight staff-level factors contributing to renal health disparities and provide a richer understanding of the organisational policy audit score.

Qualitative techniques used for analysis:

1. grounded theory techniques—using open-ended questions in interviews and surveys, studying archival data (eg, ANZDATA registry outcome data);
2. content analysis—using the presence of certain words, themes and/or concepts within the transcribed interview; and
3. narrative analysis —used to understand the differences in cases and describing context.

### Part 3: implementation

The third part of RTC involves the Community of Practice (CoP), site committee and the leadership group working together with volunteer RRT patients who come together seeking to understand the patient experience and the possible correlation to patient outcomes in the renal unit. RTC personnel will work with renal health service staff to form a working group and/or help facilitate a workshop to develop an action plan to enhance the provision of culturally safe care to First Nations renal patients and help increase the number of renal patients receiving treatment closer to home. The renal service committee will be responsible for communication at service level and the development of the intervention strategies. The establishment of the CoP may provide an ongoing mechanism via which patient and community can feedback on their experiences and suggestions which can be used by the renal service to improve service provision. Members of the CoP will include renal patients, community members and renal health practitioners. They develop a shared repertoire of resources: experiences, stories, tools, ways of addressing recurring problems—in short, a shared practice.

#### Data collection

Follow-up service-level data collection will be repeating steps I–V:

- A follow-up organisational audit (with the same tool at baseline) will be conducted a minimum of 1 year after the initial audit measurement to monitor any change in policies and procedures.
- Using the same methodology and survey tool as the initial staff survey, after a minimum of 1 year service staff will be invited to participate in a repeat staff survey.
- Additionally, ANZDATA registry data will be obtained to analyse trends and changes in practice and patient outcomes.
- Staff of participating renal services will be invited to attend a final feedback session(s)
- All patients, particularly but not exclusively First Nations patients, will be invited to at least one final feedback session at which aggregated, and service-specific study findings. Short videos and posters will facilitate communication of study findings in plain English.

Additionally, process data will be collected from the CoP. These data can include staff and consumer perspectives, planning schedules, implementation strategies, photos or videos of the activities or strategies that are implemented, and recorded reflections.

#### Data analysis

Data analysis will follow steps I–V as described in Part 2. Multi-level logistic or Poisson regression methods will be used for the analysis of the effect of interventions, clustered by the centre and analysed at the individual level, with the introduction of one or more interventions incorporated as time-dependent variables.

##### Process evaluation

Process data will be critically reviewed throughout the project to identify any barriers and facilitators to change and inform changes in project methods and processes to maintain research rigour and study integrity.

##### Mixed methods analysis

Qualitative and quantitative data will be analysed, synthesised and integrated to provide a comprehensive understanding of overall project impacts and outcomes.^50^,^52^

In summary, both patient-reported experience, as well as patient-level registry, service-level aggregate staff survey data and policy audit data will be collected to meet the objectives of the study. See table 1.

**Table 1:** Study objective and measures

Objective	Measures and tools (data collection)
To test the feasibility and appropriateness of a patient survey adapted for First Nations peoples (both in English and several First Nations languages)	Adapted video reflexive ethnography and evaluation of patient and interpreter feedback
To understand the factors associated with renal health disparities (ie, in relation to transplantation and dialysis treatment) experienced by First Nations patients compared with non-First Nations patients.	Staff survey ANZDATA registry data Organisational audit Qualitative interviews with renal service staff
To assess the level of cultural safety among renal health services and their staff from the patient experience perspective	Patient perspectives via feedback session
To identify the barriers and enablers for organisational change in relation to the provision of culturally safe care to First Nations patients with kidney failure	Community of Practice process data (see above) Qualitative interviews with renal service staff and key informant interviews
To assess and evaluate the extent to which health services change their delivery of care to First Nations patients with kidney failure following an assessment of their level of cultural safety	Community of Practice data (this can include staff and consumer perspectives, planning schedules, implementation strategies, photos or videos of the activities or strategies that are implemented, and recorded reflections.) Baseline and follow-up organisational audits Baseline and follow-up staff surveys Patient perspectives via feedback session
To assess the impact of the renal health service-based changes on health outcomes for First Nations patients with kidney failure	ANZDATA registry data

### Theoretical frameworks and principles

The RTC study is underpinned by cultural safety principles. According to the Aboriginal and Torres Strait Islander cultural safety framework,^32^ the principles of culturally safe workplace and services are respect for culture (community and individuals), equity (which requires adjustments to individual needs instead of one group) and sustaining meaningful partnerships. It is focused on First Nations expertise, self-determination and leadership: cultural safety must be led by First Nations people. Elements of cultural safety include knowledge of and respect for self and First Nations people, a commitment to redesigning organisations and systems to reduce racism and discrimination and an understanding that cultural safety is an ongoing learning journey.

Thus, cultural safety involves healthcare organisations engaging in ongoing reflection and self-awareness and holding themselves accountable for providing culturally safe care, as defined by the patients and their communities.^33^

### Study sample

Convenience and purposeful sampling will be used in the study. With all 14 invited tertiary renal services participating in the study, RTC aimed to reach an estimated 1250 renal health staff and 900 First Nations renal patients. Given delays from research moratoria due to the COVID-19 pandemic at all sites, a more targeted recruitment strategy focussing on eight sites caring for the majority of First Nations patients commencing RRT will still provide data about the care of 750 First Nations patients by an estimated 600 renal health staff.

Part 1 will be completed in four sites (Darwin, Alice Springs, Cairns and Newcastle) with between 15 and 20 patient participants per site.

#### Patient and public involvement

RTC has been developed in response to needs identified by First Nations people and communities. A local First Nations advisory group at each site will be convened to inform the study process and provide feedback to the local First Nations community. In Part 1 of the study, patient and interpreter feedback on the patient survey tool will inform its design. In Parts 2 and 3, feedback from patient and consumer groups will be incorporated into the renal health service’s cultural safety assessment to inform the development of their action plan to improve cultural safety. Local First Nations patient and consumer groups of each participating renal health service will be provided with a summary of the service’s staff survey and organisational audit findings, as well as a summary service-specific ANZDATA registry data. This feedback process will be used to stimulate reflection and discussion among patients during talking circles (as outlined in Part 2, Step V above)

### Ethics and dissemination

Ethics approval has been obtained from the Human Research Ethics Committee of the Northern Territory Department of Health and Menzies School of Health Research (NTHREC 2019-3530), the Central Adelaide Local Health Network Human Research Ethics Committee (CALHN HREC reference: 2023/HRE00209), the Aboriginal Health Council of South Australia (AHREC Protocol #: 04-23-1078), the Aboriginal Health and Medical Research Council of New South Wales (AH&MRC HREC reference: 2230/24) and the Far North Queensland Human Research Ethics Committee (FNQ HREC reference: HREC/2023/QCH/99606 (Nov ver 4)-1732). The RTC study will be conducted in accordance with the NHMRC Ethics Guidelines^53^ and AIATSIS Guidelines for Ethical Research in Australian Indigenous Studies.^54^

### Research with First Nations people

This study may potentially highlight sensitivities, biases (conscious or unconscious) and possible unequal relationships related to the provision of care to First Nations patients. We acknowledge that a power differential can operate at multiple levels: patient-renal staff, patient-renal service unit (system) and renal staff management. The formation of a study site team comprising of renal health personnel and patients is a step towards an equitable partnership (relationship) between the renal health service system, participants (patients) and community.

There will be community participation in this project, including contributions to the research design and objectives from the Aboriginal and Torres Strait Islander Alliance group and patient groups linked to the renal health services. Part 1 of the study specifically recognises that the collection of patient experience information is generally conducted in English and thus seeks to explore the acceptability, feasibility and appropriateness of its use for First Nations people who do not speak English as their first language. This study employs First Nations co-researchers fluent in the relevant language to collect this data.

### Informed consent

#### Part 1

Information will be shared about the study both in writing from plain English statement and will be explained verbally by the local researcher. Potential participants will be given time of up to a week to process the information with a key support person, which can be a family member, before giving informed consent. Consent can be verbally (recorded) or written on the consent form and needs to be witnessed. Interpreter services will be made available for participants where English is not their home language. Researchers will emphasise that participants can withdraw or say no without prejudice at any time, and this will not affect their treatment. Data collected up until the withdrawal date will be included in the study unless the participant requests the exclusion of their data.

#### Part 2

Consumers will receive written and verbal study information at least 1 week before participation in the talking circles and will be reminded about the purpose of the gathering at the start of the talking circle. At that time, group consent will be collected by research staff and participants will be offered the option to provide individual written consent. They will each sign the attendance sheet as well. A local Aboriginal language and cultural advisor will be present at each talking circle.

#### Part 3

During the CoP group consent will be collected by research staff, seeking access to video/audio recordings and photos taken during the CoP sessions and written observations of the activities implemented.

### Dissemination

Each participating renal health service will be given a report of their staff survey, organisational audit, patient group feedback and ANZDATA registry data at baseline and follow-up. Local patient and consumer groups of each renal health service will be provided with a summary of the service’s staff survey and organisational audit findings. The study’s findings will also be communicated to all the investigators on a biannual basis throughout the project.

It is anticipated that a range of presentations and journal articles will arise from the study. These will be reported nationally and internationally to patient and consumer groups as well as healthcare staff, policy makers and organisations. Communication throughout the project may include posters, newsletters, media broadcast and social media.

### Summary

The RTC study aims to improve health service delivery for First Nations people with kidney failure and has broader significance for encouraging home-based RRT modalities, including self-care dialysis and kidney transplantation. Study findings will be used to inform proposed national guidelines for the care of First Nations renal patients and help develop the infrastructure, clinical and research network needed for further interventional studies in this area. In addition, extra data collection through the ANZDATA registry about patient-centred outcomes will lead to a reshaping of registry data collection into the future (beyond the life of the project). This study will also highlight the role of the ‘social determinants of health’ related to outcomes not only for First Nations people requiring RRT but also for First Nations people with other chronic illnesses and remote-dwelling groups internationally.
