# Supplementary material for: Study protocol: Return to Country, an Australia-wide prospective observational study about returning First Nations renal patients home
Source: BMJ Open. 2024 Nov 24;14(11):e095727. doi: 10.1136/bmjopen-2024-095727 (PMC11590795; doi:10.1136/bmjopen-2024-095727)
Supplement: Supplementary file 1 [file bmjopen-2024-095727.reviewer_comments.pdf]

PEER REVIEW HISTORY

BMJ Open publishes all reviews undertaken for accepted manuscripts. Reviewers are asked to complete a checklist review form and are provided with free text boxes to elaborate on their assessment. These free text comments are reproduced below.

ARTICLE DETAILS

Title (Provisional)

Study protocol: Return to Country, an Australia-wide prospective observational study about returning First Nations renal patients home

Authors

Jones, Yomei; Truong, Mandy; Preece, Cecilia; Cass, Alan; Heerink, Fiona; McDonald, Stephen; Jones, Scott; Mallett, Andrew John; Majoni, Sandawana William; Sajiv, Cherian; Patankar, Khalil; Mulholland, Eddie; Woldeyohannes, Solomon; Lawton, Paul

This article was not externally reviewed at BMJ Open. Protocols that have been independently assessed prior to submission to BMJ Open are usually fast-tracked to publication on the grounds that further substantial changes will not be possible. This independent assessment will usually be external, independent review for both a substantial grant award from a non-commercial or government funder and ethics approval.
